# Comparing the Outcomes of Virtual Reality–Based Serious Gaming and Lecture-Based Training for Advanced Life Support Training: Randomized Controlled Trial

**DOI:** 10.2196/46964

**Published:** 2023-09-28

**Authors:** Mehmet Emin Aksoy, Arun Ekin Özkan, Dilek Kitapcioglu, Tuba Usseli

**Affiliations:** 1 Department of Biomedical Device Technology Center of Advanced Simulation and Education Acibadem Mehmet Ali Aydinlar University Istanbul Turkey; 2 Institute of Biomedical Engineering Bogazici University Istanbul Turkey; 3 Department of Medical Education, Medical Faculty Center of Advanced Simulation and Education Acibadem Mehmet Ali Aydinlar University Istanbul Turkey; 4 Vocational School for Anaesthesiology Technicians Acibadem Mehmet Ali Aydinlar University Istanbul Turkey

**Keywords:** Advanced Cardiac Life Support, virtual reality, serious game, randomized controlled trial, Advanced Life Support

## Abstract

**Background:**

Simulation-based Advanced Cardiac Life Support (ACLS) or Advanced Life Support (ALS) training for health care professionals is important worldwide for saving lives. Virtual reality (VR)–based serious gaming can be an alternative modality to be used as a part of simulation-based ALS training.

**Objective:**

The aim of this study is to investigate whether a VR-based ALS serious game module can replace classroom-based ALS lectures, the latter being part of existing conventional ALS training protocols in addition to skills training.

**Methods:**

Participants were students from Acibadem Mehmet Ali Aydinlar University’s Vocational School for Anesthesiology (N=29) randomly divided into 2 groups with 15 (conventional training group) and 14 (VR-based training group) participants each. Participants in the conventional training group had to complete the pretest consisting of multiple-choice questions at the beginning of the study. Afterward, they took part in an interactive classroom-based ALS lecture. The next step involved skills training with task trainers to teach them compression skills. Following this, the conventional training group was divided into Code Blue teams, each consisting of 5 participants for the simulation session. Two independent instructors evaluated video recordings in terms of technical and nontechnical skills. The score acquired from the manikin-based simulation session was considered the main performance indicator in this study to measure the learning outcome. A similar workflow was used for the VR-based training group, but this group was trained with the VR-based ALS serious game module instead of the theoretical lecture. The final stage of the study involved completing the posttest consisting of multiple-choice questions. A preference survey was conducted among the study participants. Mann-Whitney *U* and Wilcoxon signed-rank tests were used to analyze the 2 groups’ performances in this study.

**Results:**

The improvement in posttest results compared with pretest results was significant in the conventional training group (*P*=.002). Hands-on technical scores of the conventional training group were higher than those of the VR-based training group during manikin-based simulation, but total scores, including those for technical and crisis resource management skills, acquired from the manikin-based simulation session did not reveal any significant difference between the 2 groups. The results of the VR preference survey revealed that the majority of the participants prefer VR-based serious game–based training instead of classroom lectures.

**Conclusions:**

Although hands-on technical scores of the conventional training group during the manikin-based simulation session were higher than those of the VR-based training group, both groups’ total performance scores, including those for technical and crisis resource management skills, did not differ significantly. The preference survey reveals that the majority of the participants would prefer a VR-based ALS serious gaming module instead of lecture-based training. Further studies are required to reveal the learning outcome of VR-based ALS serious gaming.

**Trial Registration:**

ClinicalTrials.gov NCT05798910; https://clinicaltrials.gov/study/NCT05798910

## Introduction

Advanced Cardiac Life Support Course (ACLS) developed by American Heart Association (AHA) and Advanced Life Support (ALS) course developed by European Resuscitation Council (ERC) with a similar content to ACLS, aim to train healthcare professionals in managing adult patients suffering from cardiac arrest. The target group consists of medical doctors, nurses, and paramedics [1-4]. Around 1.3 million candidates take part in ACLS or ALS courses worldwide every year [5]. These training sessions have to be repeated at certain intervals depending on local regulations or institutional requirements [6].

Participants learn how to deliver high-quality cardiopulmonary resuscitation (CPR) to adult patients and to manage cardiac arrest cases. Besides technical skills, another aim of these courses is to provide training for nontechnical skills, which are essential when working as a multidisciplinary team. Blended learning techniques including didactic lecture-based, video-based learning; serious gaming; and simulation-based training modalities are used for ALS training [1,2,5].

Like in other industries, serious gaming is becoming an additional training modality for simulation-based education for healthcare over the last decade. A significant amount of time is spent in lecture-based sessions, which are generally organized before hands-on training for ALS in order to teach cardiac rhythm interpretation, drug dosage, the defibrillation procedure, and differential diagnosis [6,7]. By using serious gaming modules for this aspect of training, the educators can reduce the time spent on theoretical lectures and alleviate logistical difficulties, such as coordinating the training with a clinician from the hospital [6]. Another advantage is the ability to minimize the instructors' workload by delivering these training sessions with virtual reality (VR) modules [6]. Besides immersive features and interactivity, serious gaming modules also provide learners the opportunity to improve their skills in a safe environment anywhere and anytime they want [8-13]. PC-based, tablet-based, and VR-based serious gaming modules for health care training are available commercially. Due to their immersive effect, VR-based serious gaming modules are now widely used in health care training [6-8,14-19]. As VR hardware has already become more affordable, significant growth of VR-based learning is expected [20]. With technological developments, the new generation of VR headsets is becoming more affordable than the older ones, which cost much more and required a cable connection to a PC with an expensive graphics card to operate. The new generation of VR headsets also eliminate the huge logistical and hardware requirements for using VR. Another advantage of such a VR-based serious gaming module is the ability to be used with wireless VR headsets without the need for a PC and an expensive cable-based VR headset. Therefore, Meta Quest 2 headsets (Meta) were used in this study [21].

It is obvious that nontechnical skills are as important as technical skills when performing an ALS procedure as part of the Code Blue team [22,23]. Nontechnical skills including teamwork, resource management skills, and situational awareness are very important competencies that are required when performing an ALS procedure, as ALS is a team-based procedure [22,23]. Several serious gaming modules for ALS training are available in the market [6,24]. The existing VR-based ALS serious gaming modules are mostly focused on technical skills when calculating the total score upon final evaluation of the ALS learning outcomes. Therefore, the development of a new serious gaming module for ALS training has been decided, which can also evaluate and score nontechnical skills of the trainee in parallel with technical skills required to perform ALS. The objective of this study is to investigate whether a VR-based ALS serious game module has the potential to replace classroom-based ALS lectures, the latter being part of existing ALS training protocols in addition to skills training.

## Methods

### Study Design

This study was designed as a randomized controlled trial comparing the results of a VR-based serious gaming module and conventional training for ALS. Pretest, posttest, preference survey outcomes and hands-on training scores of the participants, which is the main performance indicator of the study, were compared.

### Recruitment

In total, 30 third-semester students of Acibadem Mehmet Ali Aydinlar University’s Vocational School for Anesthesiology volunteered to participate in this study. Simulation-based ALS training is part of their regular curriculum. The participants were randomly divided into 2 groups, with 15 participants each in the conventional (hereinafter called “group C”) and VR-based (hereinafter called the “VR group”) training groups. The VR headsets were cleaned with an antiseptic solution before each training session.

### Data Exclusion

Exclusion criteria for the study were having previously experienced VR-induced motion sickness or other medical conditions such as vertigo. One participant from the VR group was not able to attend at the study due to personal reasons and was excluded from the study.

### Statistical Analysis

Mann-Whitney *U* and Wilcoxon signed-rank tests were used to analyze the 2 groups’ performances in this study.

### VR-Based Serious Gaming Module for ALS

The serious game module for ALS has been developed for this study and named “3DMedsim ALS VR.” During the initial phase of development, the latest versions of international ALS or ACLS protocols from the AHA and ERC were reviewed together with the clinicians and software developers [1,2]. Crisis resource management (CRM) criteria adapted from those used in aviation and used for team-based training were also reviewed [22,25,26].

In the next phase, the development process of the VR-based gaming module was initiated. The VR-based serious game module developed for this study was designed to be compatible with the 2020 ALS algorithm of the ERC and AHA [1,2]. It comprises a learning management system and a learning record store (LRS), which allow users' credentials to be stored in a shared database [27-29]. The 3D visualization engine is also included and is integrated with the LRS to track the users' actions and generate experience application programming interface (xAPI) calls for each action carried out [28]. When the user triggers a predefined interaction with a significant object in the virtual environment, a xAPI event is automatically generated [27,30]. To enable this, a software library has been developed as a Unity extension, which makes the necessary xAPI web service calls to the LRS servers over the HTTP protocol. All user actions were defined in terms of actor, verb, and object parameters. The library automatically generates xAPI calls for each action, which are recorded by the LRS. The library can automatically generate xAPI calls for actions that have not been performed within a given time limit or in the proper order. Finally, the library also implements security and user authentication features required for record-keeping using the basic access authentication method included in the HTTP protocol standard.

**Figure 1:**
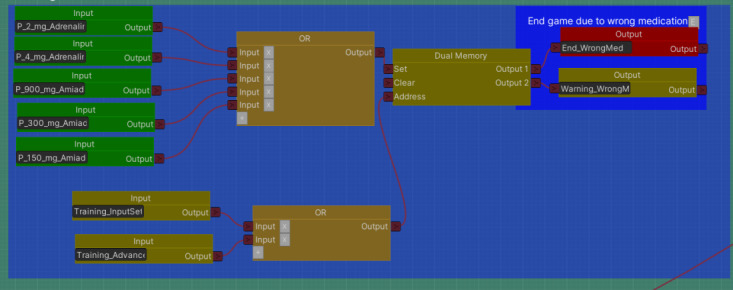
Screen capture of scenario logic flow.

To accelerate the development process and increase flexibility subsequently, a scenario building Unity plugin was developed [31]. This tool helps create and edit serious game scenarios as logic nodes outside of the virtual environment, independent from 3D objects. Thus, scenarios can be edited without altering Unity objects, which normally take the most time during the development process. The scenario building tool was only used during the development process and cannot be used by trainers to change the scenario flow during gameplay. The scenario building tool returns function calls to Unity in accordance with the logic flow, and these calls are linked to 3D objects as triggers for certain actions. As actions are triggered, they, in turn, trigger the next steps in the scenario until the serious game is over. A part of a scenario flow can be seen in Figure 1.

**Figure 2:**
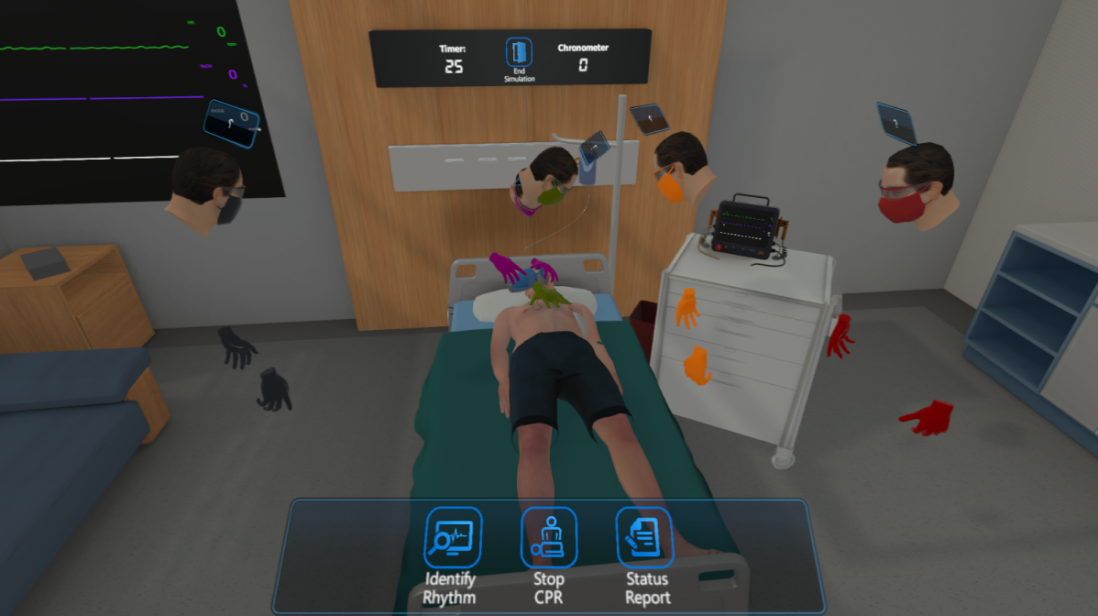
Screen capture from the virtual reality–based serious game for advanced life support.

The serious game consists of 3 different stages in multiple languages: basic training, advanced training, and a test mode. Initially, the serious game consisted of only training and test modes. However, during early testing, it became clear that a third stage was necessary, as the increase in difficulty and complexity was too much between the 2 modes. Another slightly more difficult training mode was added for this purpose. These stages can be completed in a virtual hospital or an examination room environment as seen in Figure 2.

The first stage, the basic training mode, is for beginners who wish to learn the fundamentals of the ALS algorithm. This version provides visual and audio guidance to the user at every step of the algorithm. An announcer verbally directs the user to perform specific actions, while corresponding buttons are highlighted on the interface. The user is not allowed to interact with anything that is not highlighted, and the risk of failure is thus eliminated. Additionally, time limits are removed, and the user is reminded to undertake the next step if user stays idle for a particular period.

The next stage is advanced training, which is intended for users who have completed the beginner-level training of the game. The advanced training version does not provide any visual or audio assistance, and the user is free to take any action; thus, mistakes are now possible. If users make significant mistakes or miss a time constraint, they receive a warning, but the game is not terminated.

**Figure 3:**
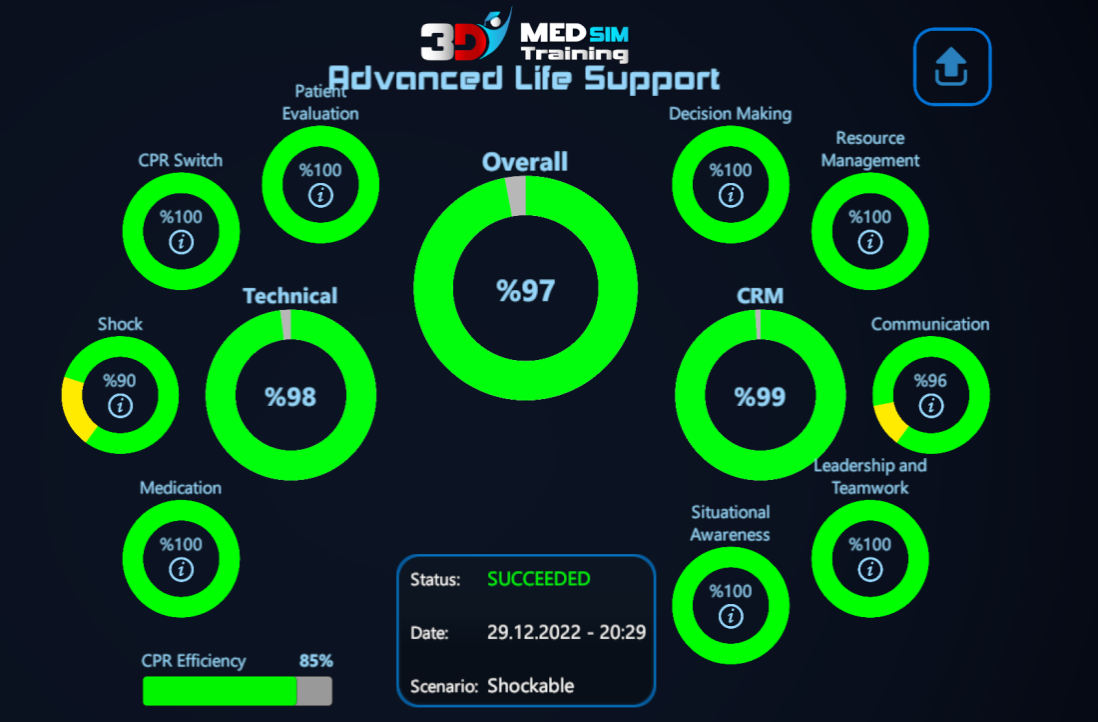
Screenshot from the serious game showing technical, crisis resource management, and total scores of a trainee. CPR: cardiopulmonary resuscitation; CRM: crisis resource management.

The third stage is the test mode. The users' actions are evaluated on the basis of their order and timing during this mode. The evaluation is carried out under 2 categories: technical and CRM skills. The scores can be accessed by using the review section. Unlike the training stages, if the user commits a significant error, such as selecting the wrong rhythm, the game terminates with a warning indicating the mistake. The technical and CRM scores are segregated as shown in Figure 3. The subcategories of these grades are specified to pinpoint the users’ errors during review.

The total score of a participant was calculated as the sum of the technical and CRM scores during the game. The maximum total achievable score was 100, that for technical skills was 70 points, and that for CRM skills was 30 points. In order to avoid risk of failure due to problems concerning the content, scenario flow, and scoring, the first version of the game was presented to clinicians for evaluation. Depending on the feedback and critiques from the content experts, changes were made. The version used in this study is the second version of the software optimized depending on user feedback.

### Study Flow

**Figure 4:**
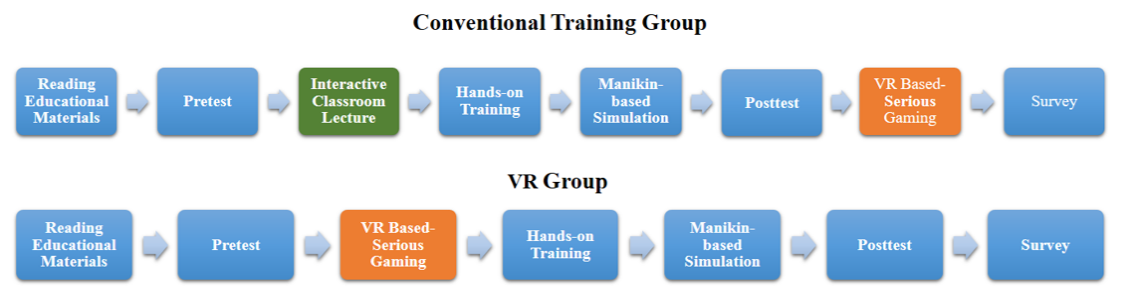
The study flow of the conventional training group and virtual reality (VR)–based training group.

The details of the study flow are shown in Figure 4.

During the initial stage of the study, all participants were asked to read the educational material sent to them. Then, both groups were asked to complete the pretest form shown in Multimedia Appendix 1.

**Figure 5:**
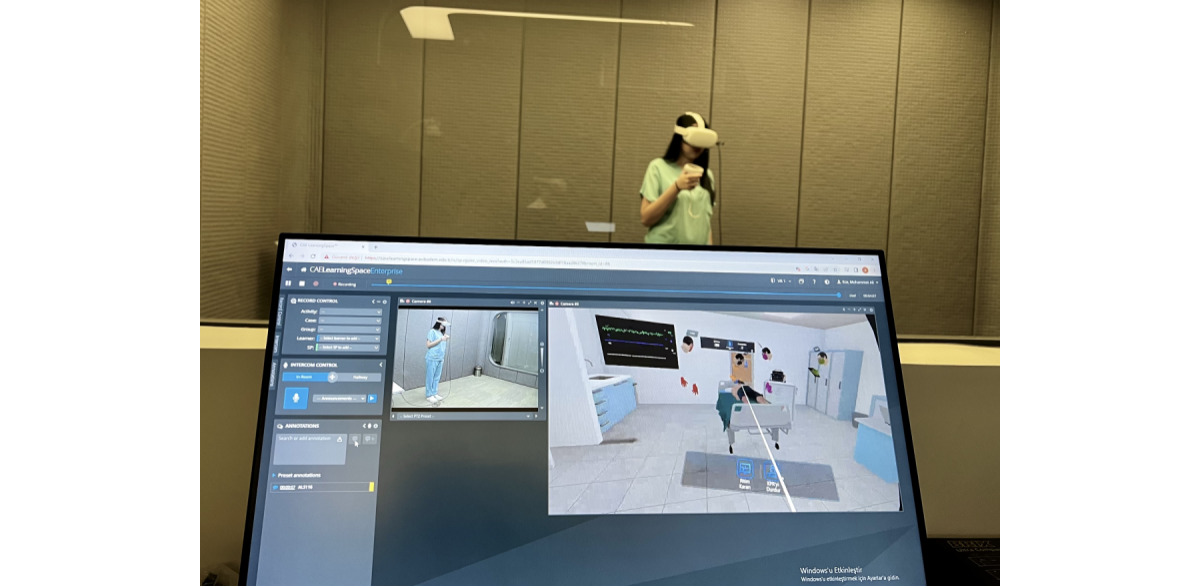
Real-time video of the trainee and virtual reality–based advanced life support serious game screen recorded during training sessions.

The pretest and posttest consisted of the same multiple-choice questions, and the test content is aligned with the curricular content required for ALS training at Acibadem Mehmet Ali Aydinlar University. This test is being used to assess of ALS training at our university and is compatible with the content of the latest version of the ERC’s training algorithm. The two-rounded Delphi technique was used with content experts when generating the pretest and posttest questionnaires. Group C participants took part in an interactive lecture with the instructors. VR group participants took part in a VR familiarization session and played 1 round of the VR beginner training mode followed by 1 round of the VR advanced training mode (Figure 5). The total time spent for these 2 rounds with VR training was equal to the time the other group spent during the classroom lecture.

**Figure 6:**
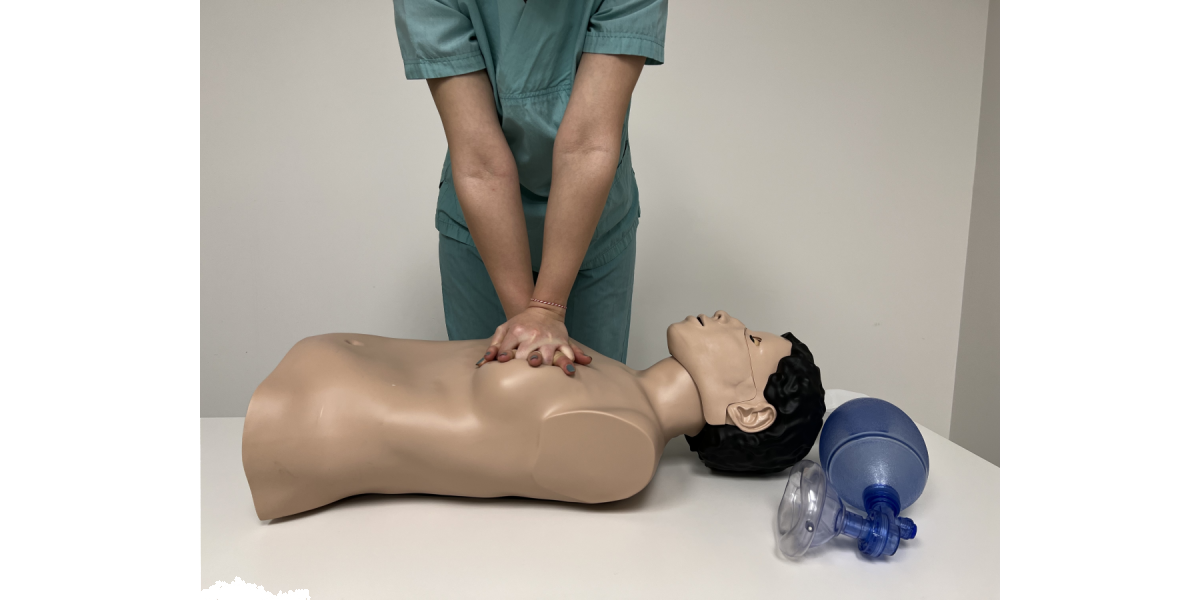
Hands-on training sessions performed using Basic Life Support manikin (CPR Lilly Pro+, 3BScientific GmbH).

Afterward, participants of both groups took part in a skills training session in order to learn effective CPR and ventilation with a CPR manikin (CPR Lilly Pro+, 3BScientific GmbH; Figure 6) [32].

**Figure 7:**
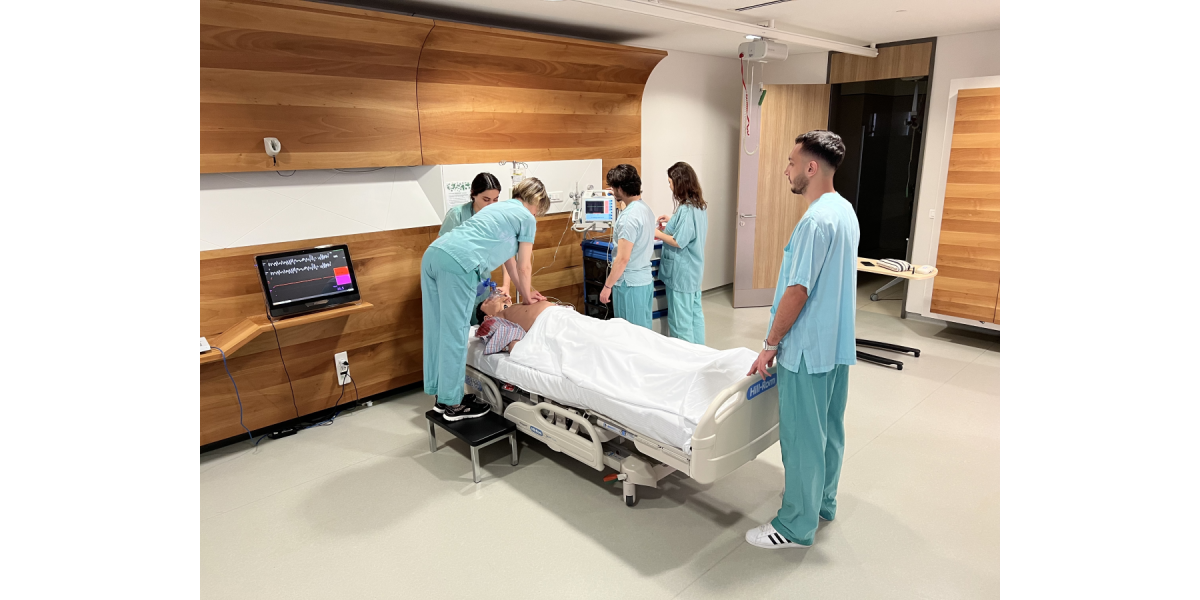
Manikin-based simulation of an advanced life support scenario with a high-fidelity patient simulator (Apollo Patient Simulator, CAE Healthcare).

**Figure 8:**
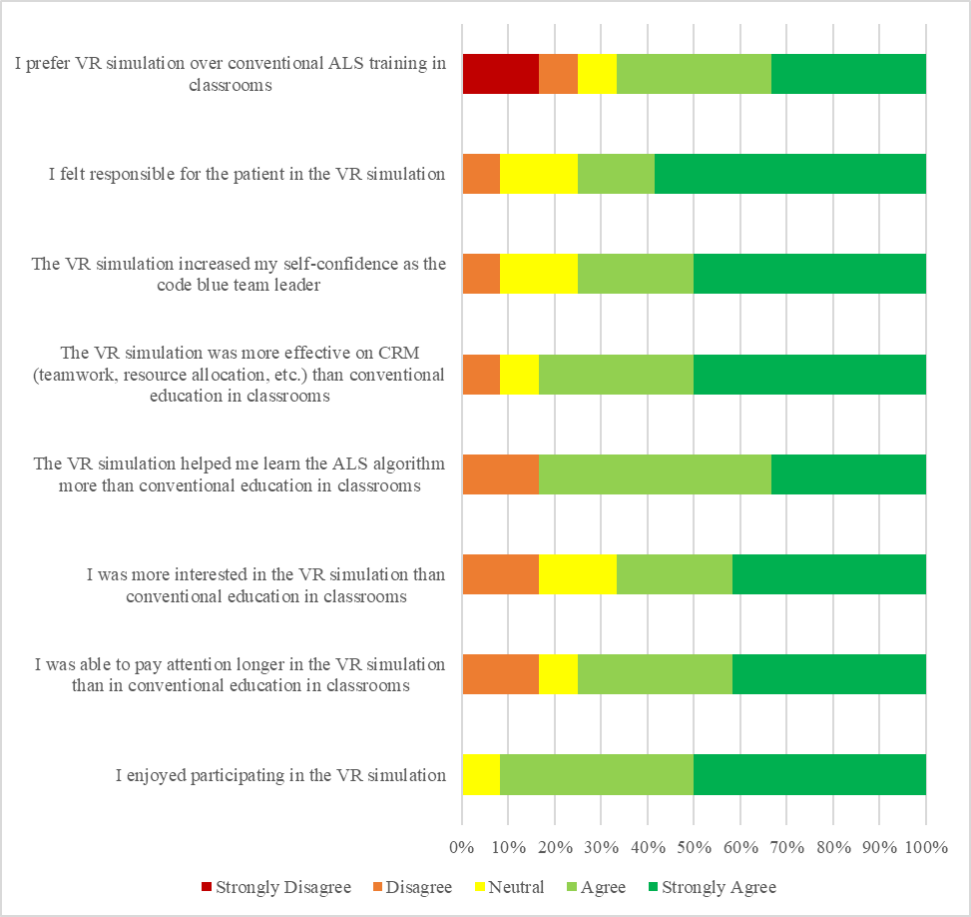
Results of the preference survey.

After the skills training, both groups took part in simulation-based ALS scenario using a patient simulator (Apollo Patient Simulator, CAE Healthcare; Figure 7) [33]. The participants were divided into Code Blue teams, each consisting of 5 participants during each simulation session. The content and flow of the scenario were identical to the scenario used for the VR-based ALS serious gaming module. The manikin-based simulation sessions of all participants were video recorded for evaluation. Two independent instructors evaluated the video-recorded performances of the participants during the manikin-based simulation scenario with the same scoring criteria used for the VR-based ALS serious gaming module. Following the manikin-based simulation session, all participants were asked to complete the posttest, which was identical with the pretest. Group C participants were given the opportunity to try the VR-based module at the end of the study; however, as the VR group participants were attending classroom-based lectures during their standard educational program, there was no need for them to take part in a classroom-based lecture at the end of the study. At the end of the study, all study participants were asked to answer the preference survey form shown in Figure 8.

### Ethical Considerations

This study has been approved by Acibadem Mehmet Ali Aydinlar University’s Ethical Committee (approval number 2022-19/04). After being informed about the content of the study, the participants signed informed consent forms. The collected data were anonymized in order to safeguard participant information. No compensation was provided to the participants.

## Results

The VR group’s pretest scores ranged from 21 to 79 (mean 43.36, SD 15.47); those of group C ranged from 21 to 79 (mean 46.53, SD 15.05). The pretest scores did not differ significantly between the 2 groups (*P*=.62). The VR group’s posttest scores ranged from 32 to 89 (mean 53.79, SD 14.01); those of group C ranged from 47 to 84 (mean 64.20, SD 9.96). The posttest scores were significantly higher in group C than in the VR group (*P*=.01). The results of the pretest and posttest are summarized in Table 1.

**Table 1:** Evaluation of pretest and posttest results (N=29).

	Total	VR^a^ Group (n=14)	Group C^b^ (n=15)	*P* value^c^
Pretest, range (median); mean (SD)	21 to 79 (45); 45 (15)	21 to 79 (44); 43.36 (15.47)	21 to 79 (45); 46.53 (15.05)	.62
Posttest, range (median); mean (SD)	32 to 89 (58); 59.17 (12.99)	32 to 89 (53); 53.79 (14.01)	47 to 84 (63); 64.20 (9.96)	.01
Difference^d^ (posttest versus pretest), range (median); mean (SD)	–11 to 42 (13); 14.17 (13.30)	–11 to 32 (10); 10.43 (11.99)	–6 to 42 (16); 17.67 (13.90)	.19

^a^VR: virtual reality.

^b^Group C: conventional training group.

^c^Mann-Whitney *U* test.

^d^Wilcoxon signed-rank test. Overall difference in pretest and posttest scores: *P*=.001; difference in pretest and posttest scores in the VR group: *P*=.02; difference in pretest and posttest scores in the group C: *P*=.002.

A comparison of the mean pretest and posttest scores is shown in Multimedia Appendix 2. There was no significant difference in the pretest scores between the 2 groups (*P*=.62). The increase in posttest scores compared to that in the pretest scores was significant in both groups (*P*=.001). The increase in the posttest scores compared to that in the pretest scores was significant in the VR group (*P*=.02). The increase in the posttest scores compared to that in the pretest scores was significant in group C (*P*=.002). A significant difference was found between the posttest scores in the 2 groups, and the scores of group C were found to be significantly higher than those of the VR group (*P*=.01).

The score of the manikin-based simulation session, which was considered the main performance indicator in this study, was evaluated with the same scoring criteria of the serious gaming module. The scores of manikin-based simulation session are summarized in Table 2. The total score of each participant consists of the CRM score and the technical score depending on their performances in the manikin-based simulation session. The maximum achievable CRM score was 30, and the maximum achievable technical score was 70. The total score was calculated as the sum of the participant’s technical and CRM scores.

**Table 2:** Evaluation of manikin-based simulation session scores (N=29).

	Total	VR^a^ group (n=14)	Group C^b^ (n=15)	*P* value^c^
CRM^d^ (maximum 30), range (median); mean (SD)	3-16 (13); 12.84 (4.20)	13-16 (14.5); 14.45 (1.23)	3-16 (13); 11.33 (5.37)	.23
Technical (maximum 70), range (median); mean (SD)	44-69 (59.3); 57.11 (8.41)	44-60.5 (58); 53.80 (7.63)	51-68.25 (63); 60.20 (8.13)	.03
Total (maximum 100), range (median); mean (SD)	57-85 (66); 69.95 (9.37)	57-76.5 (72.5); 68.25 (8.81)	64-85 (66); 71.53 (9.89)	.53

^a^VR: virtual reality.

^b^Group C: conventional training group.

^c^Mann-Whitney *U* test.

^d^CRM: crisis resource management.

The VR group’s CRM scores ranged from 13 to 16 (mean 14.45, SD 1.23); those of group C ranged 3 to 16 (mean 11.33, SD 5.37). CRM scores were not significantly different between the 2 groups (*P*=.23). Mean values of the CRM and technical scores of both groups during the manikin-based simulation session are shown in Multimedia Appendix 3. The technical scores of the VR group ranged from 44 to 60.5 (mean 53.80, SD 7.63); those of group C ranged from 51 to 68.25 (mean 60.20, SD 8.13; Multimedia Appendix 3). The technical scores of the conventional group were significantly higher than those of the VR group (*P*=.03). The VR group’s total scores during the manikin-based simulation session ranged from 57 to 76.5 (mean 68.25, SD 8.81); those of group C ranged from 64 to 85 (mean 71.53, SD 9.89; Multimedia Appendix 3). The total scores of both groups during the manikin-based simulation session do not differ significantly (*P*=.53).

At the end of the study, a preference survey was sent to the participants to evaluate VR as a learning modality. The outcome of this survey is shown in Figure 8. More than 65% of the participants agreed or strongly agreed that they would prefer VR-based serious game–based training instead of classroom-based lectures before manikin-based simulation sessions. The survey also revealed that more than 80% of the participants agreed or strongly agreed that VR-based training is more effective for them in terms of teaching CRM skills. More than 70% of the participants agreed or strongly agreed that VR-based training also increased their level of self-confidence during the manikin-based simulation scenario. More than 90% of the participants enjoyed participating VR-based gaming.

## Discussion

### Principal Results

It has been investigated whether the use of serious gaming modules could replace lecture-based sessions that are mostly organized prior to medical simulation sessions. Several studies have compared serious games with lecture-based sessions in terms of providing knowledge to the trainees [10,30,34]. Several studies have revealed that learning outcomes in the serious gaming groups were significantly better than those in the lecture groups [10,26,30]. Depending on our results, the increase in the posttest scores compared to that in the pretest scores was significant in both VR group (*P*=.02) and in group C (*P*=.002). A significant difference was found in the posttest scores between the 2 groups, and the scores of group C were significantly higher than those of the VR group (*P*=.01). A significant difference was found between the technical scores during the manikin-based simulation session between the 2 groups, and the scores of group C were higher than those of the VR group (*P*=.03). CRM scores during the manikin-based simulation session were not significantly different between the 2 groups (*P*=.23): the mean score of the VR group was 14.45 (SD 1.23), whereas that of group C was 11.33 (SD 5.37). As the maximum achievable CRM score was 30, both groups had low CRM scores, indicating that participants may require extra additional training to improve their CRM performances.

The total score of manikin-based simulation session was considered the main performance indicator of the study for measuring the participants’ learning outcome. The VR group’s total scores during the manikin-based simulation session ranged from 57 to 76.5 (mean 68.25, SD 8.81), while those of group C ranged from 64 to 85 (mean 71.53, SD 9.89). According to the statistical analysis of the 2 groups’ performances, the total scores during the manikin-based simulation session did not differ significantly (*P*=.53).

### Comparison With Prior Work

The advantages of using serious gaming as an additional training modality for simulation-based education in health care have been revealed in several studies [6,9,10,34,35]. As CPR performances of trainees deteriorate after a certain time, refresher training is required to maintain knowledge [36]. It has been shown that VR-based serious gaming for ALS is an adequate method to sustain knowledge [36,37]. Serious game–based ALS training has the potential to prevent deterioration of knowledge between updates due to its potential to be used anytime or anywhere [36]. However, for skills training, there is still the requirement to take part in simulation-based ALS training at regular intervals to maintain motor skills. A unique feature of the VR-based serious gaming module, which has been developed for this study, is that the serious game automatically scores CRM skills of the trainees apart from technical scores. When serious gaming modules for ALS training available on the market are compared with the module developed for this study, the available serious gaming modules on the market are not capable of evaluating nontechnical skills in addition to technical skills.

### Limitations

The first limitation was that only limited gameplay time for the ALS serios gaming module could be given to the VR group participants due to the busy schedule of the training center. The second limitation was that only the heads of the avatars could be displayed during the game, as wireless VR headsets available on the market can only provide limited processing power and memory capacity. The third limitation of the study was the limited number of participants. The fourth limitation was limited generalizability of our results due to this being a single-center study and recruiting only 1 type of participants.

### Conclusions

After comparing posttest and pretest outcomes, all participants improved their knowledge, but the posttest scores of group C were higher than those of the VR group. Both groups’ overall performances in the manikin-based simulation session did not differ significantly. The preference survey reveals that the majority of the participants would prefer a VR-based ALS serious gaming module instead of lecture-based education. The benefits of using a VR-based serious gaming module are the scalability of trainees’ performances and its flexibility to be used anywhere and anytime by the users. Further studies are required to reveal the learning outcomes of VR-based ALS serious gaming. Future studies could further reveal whether the performance of the VR group may improve if more time were given for users to interact with the VR-based serious game.

## Appendix

Multimedia Appendix 1 The pre-test and post-test questionnaire used to evaluate the knowledge level of the participants.

Multimedia Appendix 2 Distribution of pre-test and post-test scores.

Multimedia Appendix 3 Mean scores of the VR and conventional group acquired from manikin-based simulation.

Multimedia Appendix 4 CONSORT E-Health Checklist.

## Abbreviations

ACLS: advanced cardiac life support
AHA: American Heart Association
ALS: advanced life support
CPR: cardiopulmonary resuscitation
CRM: crisis resource management
ERC: European Resuscitation Council
LRS: learning record store
VR: virtual reality
xAPI: Experience Application Programming Interface

## Data Availability

The data sets generated during or analyzed during this study are available from the corresponding author on request.
