# Supplementary figures and images for: Comparing the Outcomes of Virtual Reality–Based Serious Gaming and Lecture-Based Training for Advanced Life Support Training: Randomized Controlled Trial
Source: JMIR Serious Games. 2023 Sep 28;11:e46964. doi: 10.2196/46964 (PMC10570891; doi:10.2196/46964)

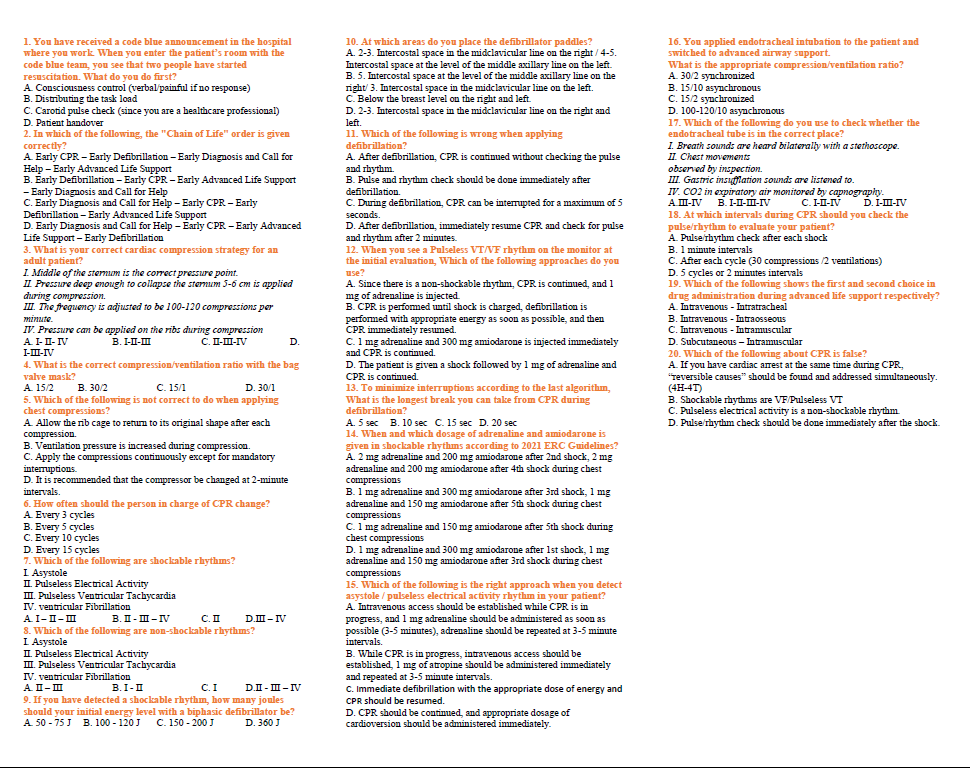

Supplement: Multimedia Appendix 1 [file games_v11i1e46964_app1.png]

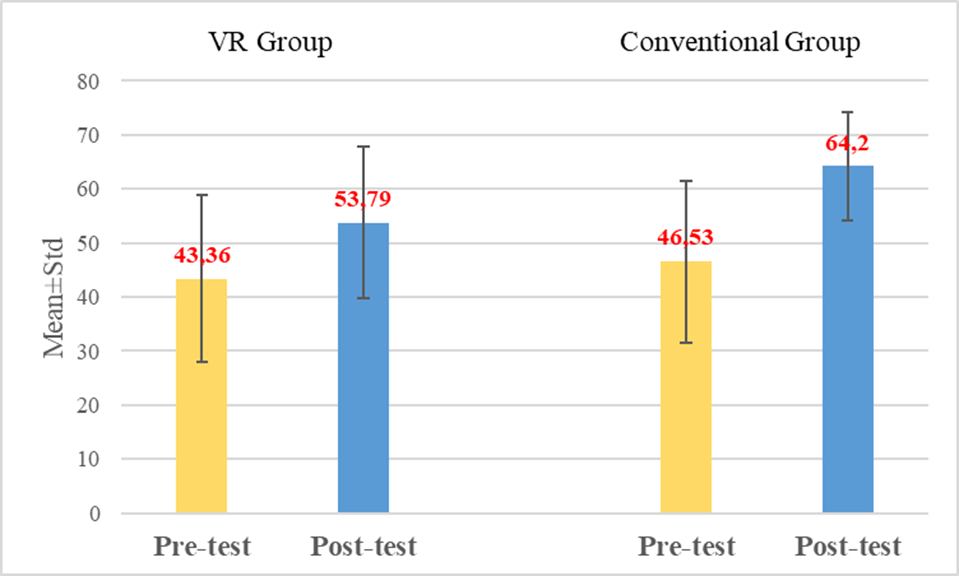

Supplement: Multimedia Appendix 2 [file games_v11i1e46964_app2.png]

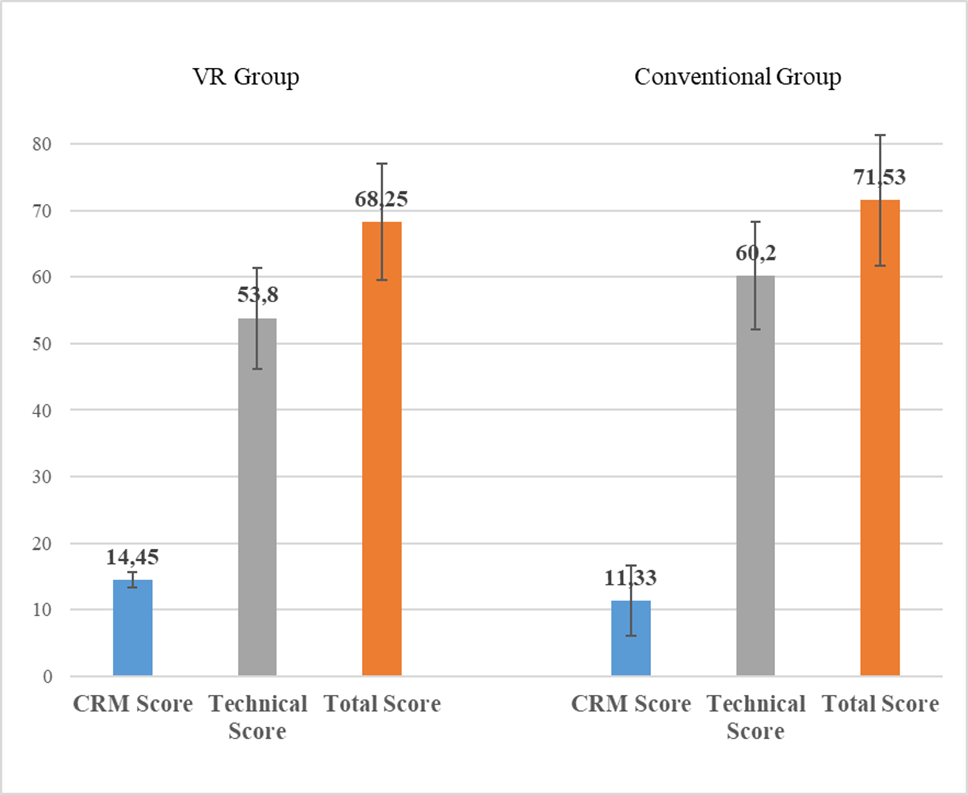

Supplement: Multimedia Appendix 3 [file games_v11i1e46964_app3.png]
